# Task-dependent contribution to edge-based versus region-based texture perception

**DOI:** 10.1038/s41598-024-68976-6

**Published:** 2024-08-02

**Authors:** Elena Gheorghiu, Cassandra Diggiss, Frederick A. A. Kingdom

**Affiliations:** 1 Department of Psychology, University of Stirling, Stirling, FK9 4LA Scotland, UK; 2 McGill Vision Research, Department of Ophthalmology and Visual Sciences, McGill University, Montreal, Canada

**Keywords:** Texture, Edge, Region, Segmentation, Contrast, Orientation, Luminance, Human behaviour, Object vision, Pattern vision, Visual system

## Abstract

Texture segregation studies indicate that some types of textures are processed by edge-based and others by region-based mechanisms. However, studies employing nominally edge-based textures have found evidence for region-based processing mechanisms when the task was to detect rather than segregate the textures. Here we investigate directly whether the nature of the task determines if region-based or edge-based mechanisms are involved in texture perception. Stimuli consisted of randomly positioned Gabor micropattern texture arrays with five types of modulation: orientation modulation, orientation variance modulation, luminance modulation, contrast modulation and contrast variance modulation (CVM). There were four modulation frequencies: 0.1, 0.2, 0.4 and 0.8 cpd. Each modulation type was defined by three types of waveforms: sinewave (SN) with its smooth variations, square-wave (SQ) and cusp-wave (CS) with its sharp texture edges. The CS waveform was constructed by removing a sinewave from an equal amplitude square-wave. Participants performed two tasks: detection in which participants selected which of two stimuli contained the modulation and discrimination in which participants indicated which of two textures had a different modulation orientation. Our results indicate that threshold amplitudes in the detection task followed the ordering SQ < SN < CS across all spatial frequencies, consistent with detection being mediated by the overall energy in the stimulus and hence region based. With the discrimination task at low texture spatial frequencies and with CVM textures at all spatial frequencies the order was CS ≤ SQ with both < SN, consistent with being edge-based. We modeled the data by estimating the spatial frequency of a Difference of Gaussian filter that gave the largest peak amplitude response to the data. We found that the peak amplitude was lower for detection than discrimination across all texture types except for the CVM texture. We conclude that task requirements are critical to whether edges or regions underpin texture processing.

## Introduction

Texture is an integral feature of the natural world, providing information about the structure of natural and synthetic objects and surfaces. Textures can vary across space in many visual dimensions such as orientation, spatial frequency, luminance, contrast, motion and depth, and such variations (or modulations) can provide important information about the location of objects^1–3^ and the shapes of textured surfaces^4–7^.

One issue that has engaged vision scientists interested in texture perception is whether texture processing primarily utilizes the information at texture edges or instead the regions between texture edges. Answering this question is complicated by virtue of the fact that there are different dimensions of texture variation (see above), different types of edge (smooth, sharp, cusp) and different types of task (detection, discrimination, effortless segmentation). Thus, what is edge-based and what is regions-based may depend on any one or other or more of the above factors.

A handful of studies have attempted to address this question directly by examining edge detection mechanisms using first order (luminance, colour) or second order (orientation, contrast) modulated stimuli. Studies examining first-order edge detection mechanisms have focused on detection of noisy luminance edge stimuli^8^, step edges embedded in brown noise^9^ and luminance^10^ or chromatic^11^ blurred edges. Evidence from these studies indicate that detection is mediated by a diversity of filters with different receptive field shapes and sizes^8^. Other studies have examined second-order boundary detection for textures defined by orientation^12–15^ or contrast^16–18^ and which cannot be detected by mechanisms sensitive to first-order boundaries^19,20^.

In one such study, by Gurnsey and Laundry^14^, required observers to select an orientation-defined texture from one of four quadrants in a spatial four-alternative forced-choice task. They found that smoothing the border between the quadrants or introducing a gap between them had only a moderate effect on performance, concluding that while both edge- and region-based mechanisms are involved in texture discrimination the region-based mechanism is the more important. Wolfson and Landy^15^ later showed that two texture regions differing in mean element orientation were most easily discriminated when they abutted. However, when the textures differed in the standard deviation of the orientation distribution (termed here orientation-variance-modulated), performance was similar for abutting and separated textures. Their study suggests that an important factor determining whether texture discrimination is edge- or region-based is the type of texture modulation.

Several texture perception studies have measured detection using textures periodically modulated along a particular dimension. In these studies, the texture detection task involves selecting the forced-choice interval containing the modulated as opposed to unmodulated texture. The dependent variable is invariably the amplitude of texture modulation required to reach threshold^17,18,21–26^. Of these studies the one by Kingdom and Keeble^23^ addresses most closely the issue addressed here, in that it considered the relative detectability of abrupt versus smooth texture modulations. Using orientation-defined textures Kingdom and Keeble measured modulation amplitude thresholds for sinewave (SN), square-wave (SQ) and missing-fundamental (MF) wave patterns across a range of modulation spatial frequencies. The sinusoidal modulation exemplified smooth texture variations, the square-wave abrupt texture variations and the missing fundamental both abrupt and smooth texture variations. Kingdom and Keeble^23^ found a clear ordering of sensitivity SQ > SN > MF and showed that the detection of all three types of waveform could be modelled by a Fourier-energy-sensitive mechanism that resembled the operation of a single linear filter. Their study showed that what mattered was the overall texture energy in the stimulus, suggesting that detection was region based. Using contrast modulated stimuli, DiMattina and Baker^16^ measured modulation thresholds for both the detection and discrimination of boundary orientation and showed that participants use spatial information in different ways in the two tasks, making use of ‘region-based' processing for detection and ‘edge-based’ processing for discrimination. The above-mentioned studies highlight the need for a comparison of different types of texture modulation, and different tasks.

In this communication, we have considered the task-dependency of various types of texture modulation, texture waveform types and modulation spatial frequency, for two tasks: detection and discrimination. The results have helped refine our understanding of the conditions favouring whether texture processing is edge- or region-based.

## Methods

### Participants

The three authors acted as participants, though one of them was naive as to the purpose of the experiments when tested. All had normal or corrected-to-normal visual acuity. Participants gave their written informed consent prior to taking part and were treated in accordance with the Declaration of Helsinki (2008, version 6). All research procedures were approved by the Research Institute of the McGill University Health Centre (RI-MUHC) Ethics Board and by the General University Ethics Panel (GUEP) at the University of Stirling.

### Stimuli—generation and display

The stimuli were generated by a ViSaGe MKII video-graphics card (Cambridge Research Systems Ltd., UK) and presented on a gamma-corrected 20-in ViewSonic Professional Series PF817 cathode ray tube (CRT) monitor (ViewSonic, Brea, CA, USA) with spatial resolution of 1024 × 768 and refresh rate of 85 Hz. Monitor luminance was gamma-corrected after calibration with an Optical OP200E photometer. All stimuli were presented in the center of the monitor on a mid-grey background with mean luminance of 47.2 cd/m^2^. Viewing distance was 100 cm. Stimuli were generated and data collected using psychophysics software written in C/C++ containing embedded ViSaGe routines.

**Figure 1:**
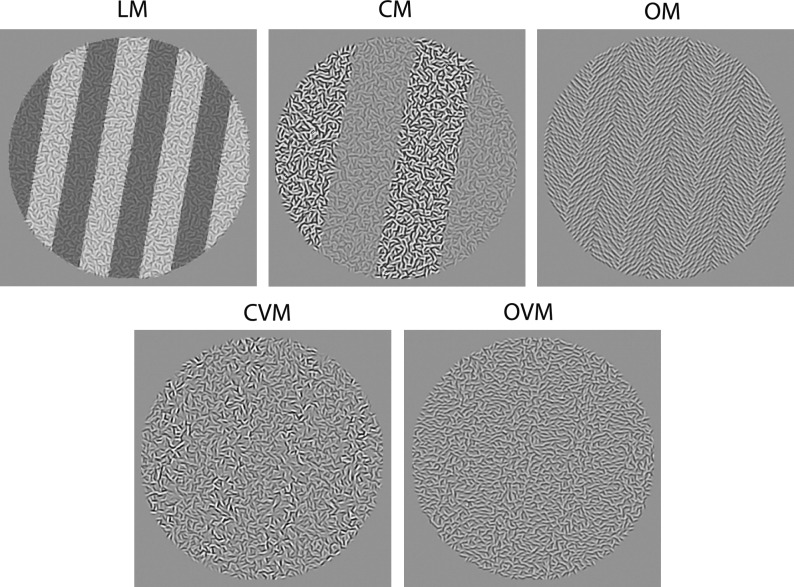
Example textures consisting of quasi-randomly positioned Gabor micropatterns arranged to produce five types of modulation: luminance modulation (LM), orientation modulation (OM), contrast modulation (CM), orientation variance modulation (OVM) and contrast variance modulation (CVM). All modulations in the figure are square wave.

Textures were 10 deg in diameter and consisted of 2500 quasi-randomly-positioned Gabor micropatterns according to five types of modulation: luminance modulation (LM), orientation modulation (OM), contrast modulation (CM), orientation variance modulation (OVM) and contrast variance modulation (CVM). Example textures are shown in Fig. 1. The Gabors were all odd-symmetric with a luminance spatial frequency of 6 cycles per degree (cpd), a bandwidth at half-height of 1.5 octaves and an envelope diameter of 5 standard deviations (SDs). Gabors were randomly positioned with the constraint that adjacent Gabors were a minimum of 1.7 SDs apart. Gabor orientations were selected from 1440 templates equally distributed across a 360 deg range, resulting in an orientation precision of 0.25 deg.

**Figure 2:**
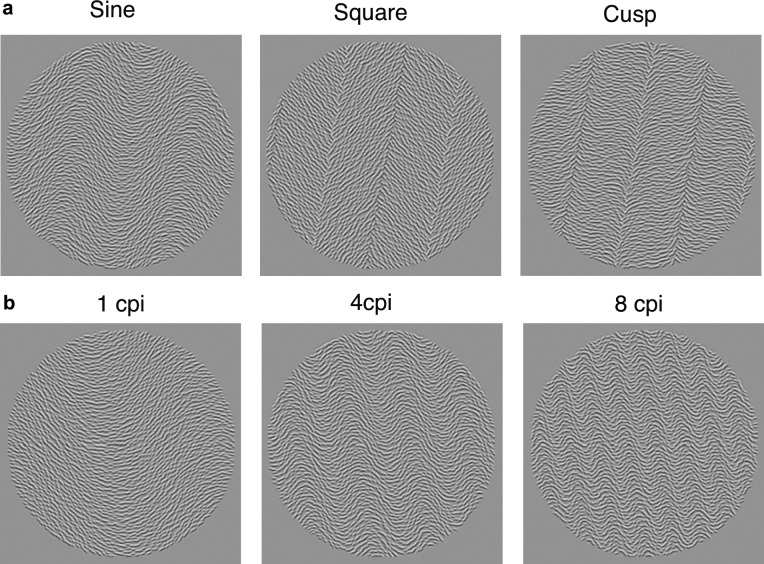
Example orientation-modulated textures. (**a**) Three types of modulation waveform: sinewave (Sine), square-wave (Square) and cusp-wave (Cusp), all at 2 cycles-per-image (cpi) or 0.2 cycles-per-deg (cpd), and (**b**) with different modulation waveform frequencies: 1, 4 and 8 cpi, corresponding to 0.1, 0.4 and 0.8 cpd.

Similar to the study by Kingdom and Keble^23^ described earlier, each texture modulation type was defined by three types of waveform: sinewave (SN), square-wave (SQ) and cusp-wave (CS), as shown for the OM texture in Fig. 2a. The cusp waveform was constructed by removing an equal-amplitude sinewave from a square-wave. The CS stimulus is similar in profile to the well-known missing-fundamental (MF) waveform^23^, formed by subtracting from a square wave its fundamental harmonic component which is 4/π or 1.273 time the amplitude of the square-wave itself. The reason we chose a CS rather than MF waveform is that the removal of an equal-amplitude SN from a SQ to make a CS allows for a more direct comparison of the amplitude thresholds for the three types of waveform. However, this is not an important constraint since the model described below transforms the stimulus amplitudes in whatever form to best fit the data. As the figure shows, the SN textures have only smooth variations, the SQ texture sharp variations with uniform regions in between and the CS waveform sharp variations and non-uniform regions in between. We used four modulation spatial frequencies: 0.1, 0.2, 0.4 and 0.8 cpd, corresponding to 1, 2, 4 and 8 cycles-per-image (cpi) (see Fig. 2b). Thus, given five types of texture modulation, three modulation waveforms and four spatial frequencies there were a total of 60 conditions for each task.

The LM textures were constructed by combining two components: a Gabor texture and a LM grating. The two components were presented on separate pages of video memory that alternated at 120 Hz. The Gabor contrasts on one video page were sinusoidally modulated with an amplitude of 0.333 and mean of 0.666 resulting in a peak-to-trough contrast ratio of 3 and an amplitude/mean texture contrast of 0.5. The LM grating displayed on the other video page had a Michelson contrast of 0.5, resulting in a peak-to-trough luminance ratio of 3. These two components were combined spatially in-phase to simulate the shading of a uniform-in-contrast texture^27^. Due to the alternation of the two video pages all contrasts reaching the eye were halved.

In the OM textures Gabor contrast was 0.333 with orientations modulated around a mean of 90 deg (horizontal). The CM textures were constructed from Gabors whose contrasts were modulated around a mean of 0.333. The OVM textures were modulated in orientation variance around a mean orientation of 90 deg, and the CVM textures were modulated in contrast variance around a mean contrast of 0.333.

### Procedure—detection versus discrimination

**Figure 3:**
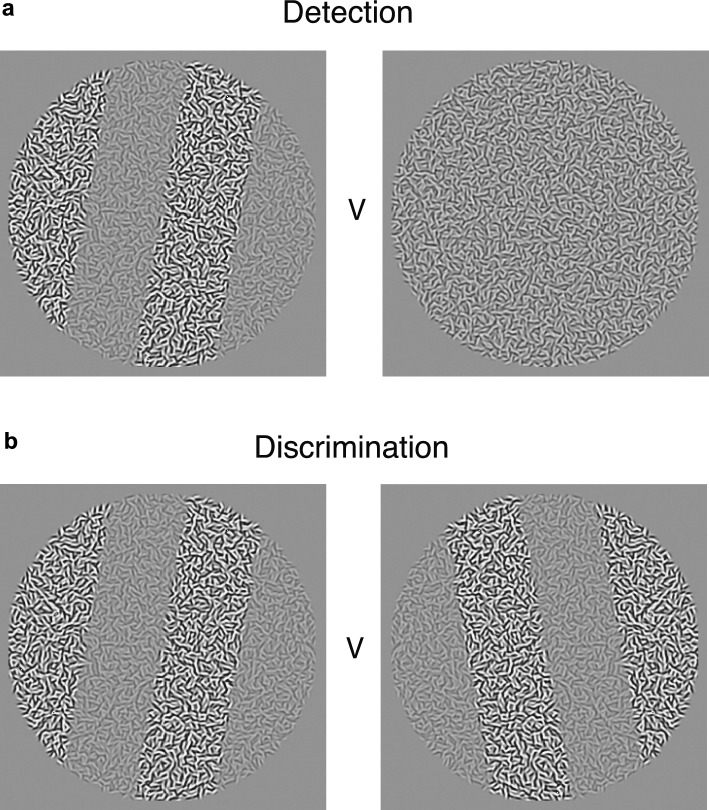
Two-interval forced-choice design for (**a**) detection and (**b**) discrimination. In the detection task participants selected the interval containing the modulation. In the discrimination task participants selected the interval with the leftward-oriented bars.

For each type of texture, a two-interval forced-choice (2IFC) design was employed to measure modulation amplitude thresholds for both detection and discrimination. All participants performed both texture tasks (Fig. 3).

*In the detection task*, the test stimulus was a modulated texture whose texture bar orientations were randomly selected from two values: ± 5 deg for OM and LM, ± 8 deg for CM and ± 10 deg for OVM and CVM. These values were the same as those selected for the discrimination task, as described below, but were different for the different texture types in order to roughly equate performance across texture type in the discrimination task, as determined by pilot studies. Modulation phase was randomized on each trial. The test stimulus was randomly presented in one of the two intervals with the other interval containing a zero-amplitude stimulus (Fig. 3a). Each stimulus was presented for 500 ms separated by an inter-stimulus interval (ISI) of 500 ms. All stimuli were presented in a raised cosine envelope with an exposure duration of 500 ms to minimize the presence of sharp temporal luminance transients. The task for the participant was to indicate by a key press which of the two intervals contained the modulation. Following each response there was an inter-trial-interval of 500 ms prior to the next stimulus presentation, enabling participant control over stimulus timing.

For each condition, we varied the amplitude of the modulation using a three-up, one-down staircase procedure with multiplicative step changes. The initial test amplitude was set to a random value within a small range above threshold. For the first five trials of each staircase the step size was 1.6 and thereafter 1.3. The staircase converged on the 79% correct threshold level and was terminated after 10 reversals. The geometric mean stimulus value of the last 8 reversals was used as the threshold. Each participant collected between 3 and 6 thresholds for each condition (i.e. combination of type of modulation, waveform and modulation frequency). The means and standard errors of these thresholds were then calculated, and these are the thresholds and error bars shown in all graphs.

*In the discrimination task,* the two intervals contained texture bars with left- or-right-oblique orientations that were the same as the orientation pairs used in the detection task. Participants were required to indicate the interval containing the left-oblique orientation (Fig. 3b). Otherwise, the protocol was the same as for the detection task.

## Results

### Experimental data

#### OM and OVM textures

**Figure 4:**
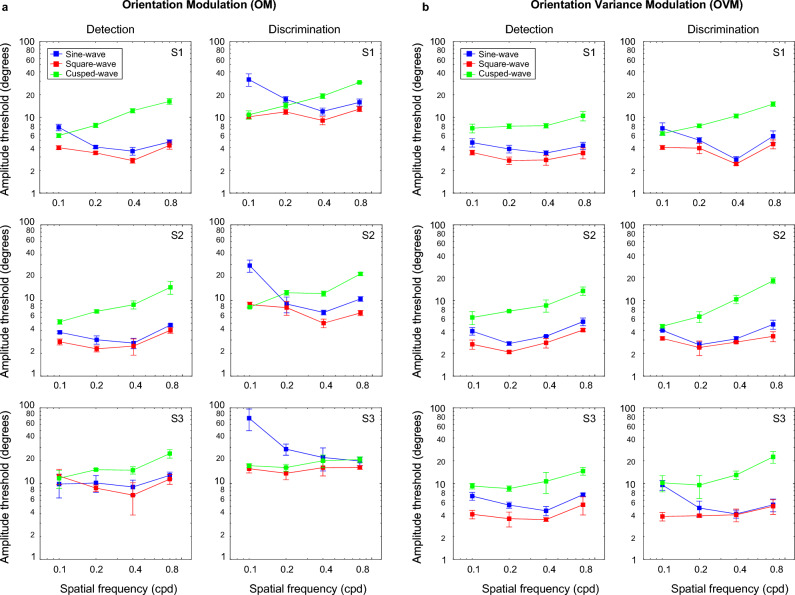
Amplitude modulation thresholds and standard errors obtained with (**a**) orientation modulated (OM) and (**b**) orientation-variance modulated (OVM) textures are shown as a function of modulation spatial frequency for sinewave (blue), square-wave (red) and cusp-wave (green) modulations, for both detection (left panels) and discrimination (right panels) tasks and for three participants S1–S3.

Figure 4 shows amplitude thresholds obtained with orientation modulated (a) and orientation-variance modulated (b) textures as a function of the spatial frequency of the modulation waveform for the sinewave (blue), square-wave (red) and cusp-wave (green) modulations for both detection (left) and discrimination (right) tasks.

For the *detection task*, the OM and OVM texture (left panels in Fig. 3a,b) results indicate lowest thresholds for the square-wave (SQ), followed by sinewave (SN) and cusp-wave (CS), thus following the ordering SQ < SN < CS. A similar pattern was also obtained for the OVM discrimination task, with OVM thresholds being of comparable magnitude in both tasks. This SQ < SN < CS result would be expected if all the texture energy available was used for detection, therefore suggesting that the OM detection and OVM detection and discrimination tasks involved region-based mechanisms. For both OM and OVM textures and both tasks, the CS thresholds increased with the spatial frequency of modulation, while SQ and SN thresholds were comparable, showing only a modest increase, if any.

However, for the *discrimination task* with the OM texture, at least at low texture spatial frequencies, threshold amplitudes obtained with SQ and CS modulations were comparable and lower than the threshold amplitudes obtained with SN modulation (i.e., they followed the order SQ ≤ CS < SN). This suggests that texture edges were the more salient features. Thus, at low spatial frequencies of modulation, the discrimination task employed edge-based mechanism. At medium and high spatial frequencies of texture modulation, the two tasks produced comparable pattern of results for OM textures, although with slightly higher thresholds for discrimination.

### LM textures

**Figure 5:**
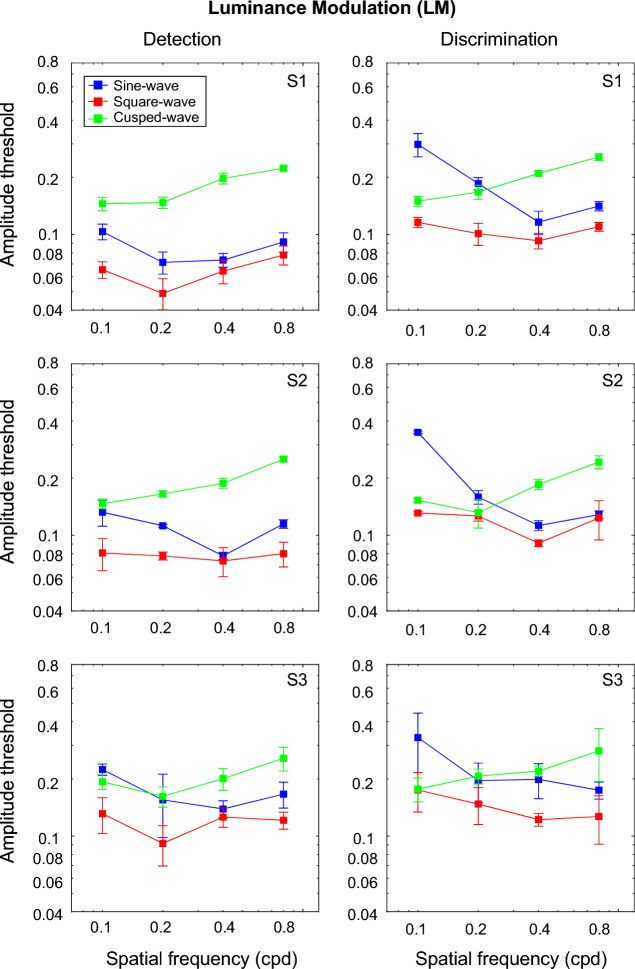
Amplitude modulation thresholds obtained with luminance-modulated (LM) textures are shown as a function of the spatial frequency of the modulation waveform for the sinewave (blue), square-wave (red) and cusp-wave (green) modulations for both detection (left panels) and discrimination (right panels) tasks.

Figure 5 shows amplitude thresholds obtained with luminance modulated textures as a function of the spatial frequency of the modulation waveform for the sinewave (blue), square-wave (red) and cusp-wave (green) modulations for both detection (left) and discrimination (right) tasks.

The LM texture results are similar with the OM textures (Fig. 4a). Specifically, the *LM detection* results follow the ordering SQ < SN < CS suggesting that LM detection task was region-based. For the LM *discrimination task*, at low texture spatial frequencies, the rule of SQ ≤ CS < SN threshold amplitudes was found, suggesting that the texture edges were more salient features and thus, the discrimination task was edge-based. As with OM texture, the CS thresholds obtained with LM textures increased with the spatial frequency of modulation, while at medium and high spatial frequencies of texture modulation, both tasks produced comparable results, although with slightly higher thresholds for discrimination.

### CM and CVM textures

**Figure 6:**
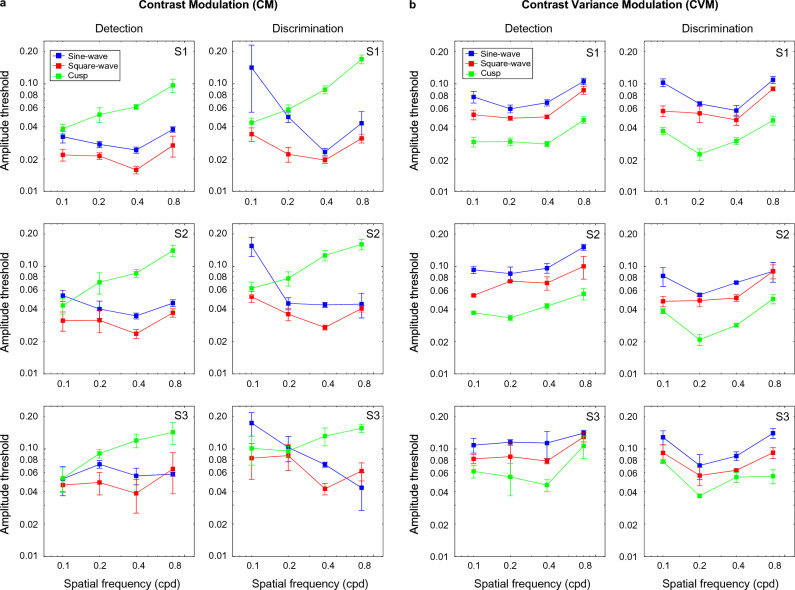
Amplitude modulation thresholds obtained with (**a**) contrast modulated (CM) and (**b**) contrast-variance modulated (CVM) textures are shown as a function of the spatial frequency of the modulation waveform for the sinewave (blue), square-wave (red) and cusp-wave (green) modulations and for both detection (left panels) and discrimination (right panels) tasks.

Amplitude thresholds obtained with contrast modulated (a) and contrast-variance modulated (b) textures are shown in Fig. 6 as a function of the spatial frequency of the modulation waveform for sinewave (blue), square-wave (red) and cusp-wave (green) modulations, for both detection (left) and discrimination (right) tasks. Figure 6 indicate a different pattern of results for CM and CVM textures (compare Fig. 6a,b). *For the CM textures* (Fig. 6a), amplitude detection and discrimination results show a similar trend to those obtained with OM and LM textures (i.e., *at low spatial frequencies*, detection thresholds are consistent with the ordering SQ < SN < CS suggesting that the task was region-based, while discrimination thresholds follow the order SQ ≤ CS < SN indicating that the task was edge-based. As before, the CS thresholds increased with the spatial frequency of modulation, while at medium and high spatial frequencies of texture modulation, the two tasks produced comparable results for CM.

*For the CVM textures*, thresholds were lowest for the cusp-wave, followed by square-wave and sinewave modulation, thus following a hierarchy of performance CS < SQ < SN, which suggests that the task was edge-based. Thresholds were also comparable across different spatial frequencies of modulation, although there was a modest improvement at intermediate spatial frequencies which was mainly present in the discrimination task (see u-shaped curves in Fig. 9b right panel). Overall, the trend in the data pattern for SN and SQ was similar to CS but shifted towards higher amplitude threshold values.

### Single filter/channel peak amplitude model

To understand the degree to which potential edge- versus region-based mechanisms mediate the variations in thresholds across conditions, we fitted a one-dimensional (1D) Difference of Gaussian (DoG) filter simultaneously to all three types of waveform across spatial frequency, for each type of texture, task and participant. It is important to note from the outset that the filter model is not meant to represent physiological reality. Rather it should be considered to be the computational equivalent of an array of filters selective for texture type and spatial frequency (e.g. see Kingdom and Keeble^23^), and a convenient method for reducing the data to a single parameter (see below) that captures the relative scale dependency of the detection and discrimination tasks.

The objective of the model is to find a DoG filter shape that best fits the measured sensitivities to one dimensional versions of the triplet of waveforms (SN, SQ, CS) for each type of texture, task and participant. The model predictions were obtained by convolving the filter with the triplet of waveforms across modulation spatial frequency and adjusting the filter parameters to minimize the difference between the convolution outputs and the measured sensitivities to the triplets, which were calculated as 1/threshold amplitude. Once the filter was fitted, we calculated the spatial frequency (SF) that produced the maximum amplitude response of the filter. We suggest that this parameter best captures the relative degree to which the task is edge- or region-based, with a relatively low peak-amplitude SF indicating that the task is more region-based and relatively high peak-amplitude SF indicating that the task is more edge-based.

The DoG filter is given by:

1 $$DoG= \frac{\left(\alpha \right)}{\gamma \cdot {\left(2\pi \right)}^{0.5}}\cdot {exp}^{\left(\frac{-{x}^{2}}{2\cdot {\gamma }^{2}}\right)}- \frac{\left(\alpha \cdot \beta \right)}{\gamma \cdot \delta \cdot {\left(2\pi \right)}^{0.5}}\cdot {exp}^{\left(\frac{-{x}^{2}}{2\cdot {\left(\gamma \cdot \delta \right)}^{2}}\right)}$$

where the free parameters are: *α* the centre gain, *β* the ratio of gains of surround to centre, *γ* the centre standard deviation (SD) and *δ* the ratio of surround to centre SDs. For each set of fitted parameters [*α β γ δ*] we determined the filter spatial frequency that gives the peak amplitude response, as given by:

2 $${SF}_{at peak amplitude}={\left(\left(log\left(\propto \right)+log\left({\gamma }^{2}\right)-log\left(\propto \cdot \beta \right)-log\left({\left(\gamma \cdot \delta \right)}^{2}\right)\right)/\left(2\cdot {\pi }^{2}\left({\gamma }^{2}-{\left(\gamma \cdot \delta \right)}^{2}\right)\right)\right)}^{0.5}\cdot w$$

where *w* is the width of the filter that enables SF at peak amplitude to be given in cycles per image and was set to 256. As a check we also determined the value of SF at peak amplitude after subjecting the filter to a discreet Fourier transform (DFT). We refer to the equation model as the ‘analytical’ model and the DFT model by its name.

The initial filter guess parameters [*α*, *β*, *γ*, *δ*] was set to [100, 1, 4, 6] were set for all participants, types of texture and task. Note that the surround to center gain ration (*β*) parameter when fitted will tend to be close to 1 to make the filter more-or-less d.c.-balanced, that is to have the gains of center and surround similar in order to avoid signal bias/ error. Given five modulation types, three participants and two tasks there were 30 estimates of SF at peak amplitude. The best fits to each triplet of data was achieved by minimizing the sum of squared differences between model and data using the PAL-*minimise* function in the Palamedes toolbox^28^, which is similar to Matlab’s *fminsearch* function but in our experience less prone to instability.

**Figure 7:**
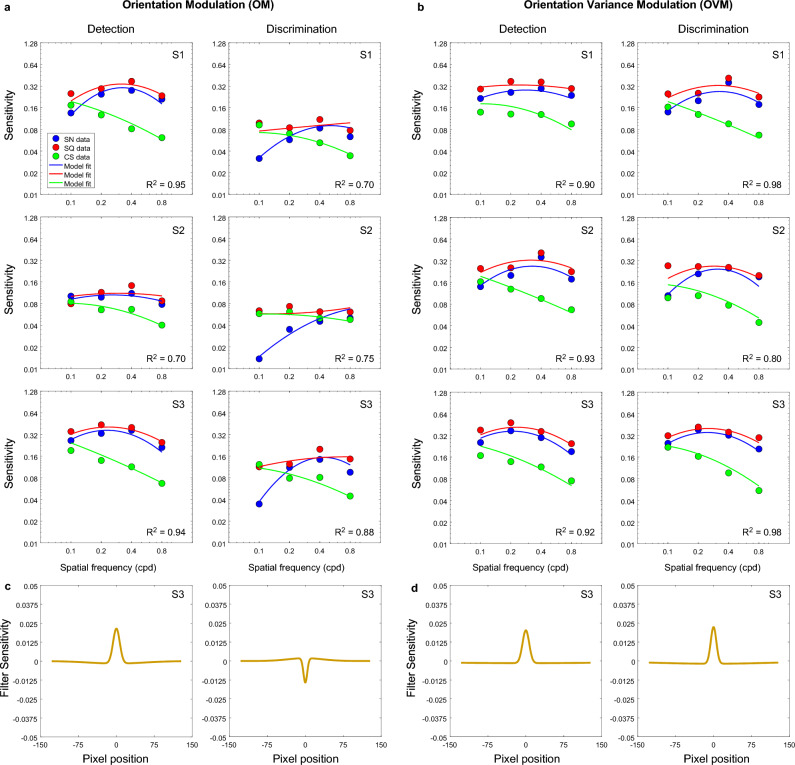
(**a**, **b**) Model fits (continuous lines) to sensitivity data (dots)(not thresholds as in previous figures) for each triplet waveforms: sinewave (blue), square-wave (red) and cusp-wave (green) for the (**a**) orientation modulated, OM, and (**b**) orientation variance modulated, OVM, texture types for both detection (left panels) and discrimination (right panels) tasks. Example corresponding shapes of the model filter (filter sensitivity) for one participant for OM (**c**) and OVM (**d**) for both detection (left panels) and discrimination (right panels) tasks.

**Figure 8:**
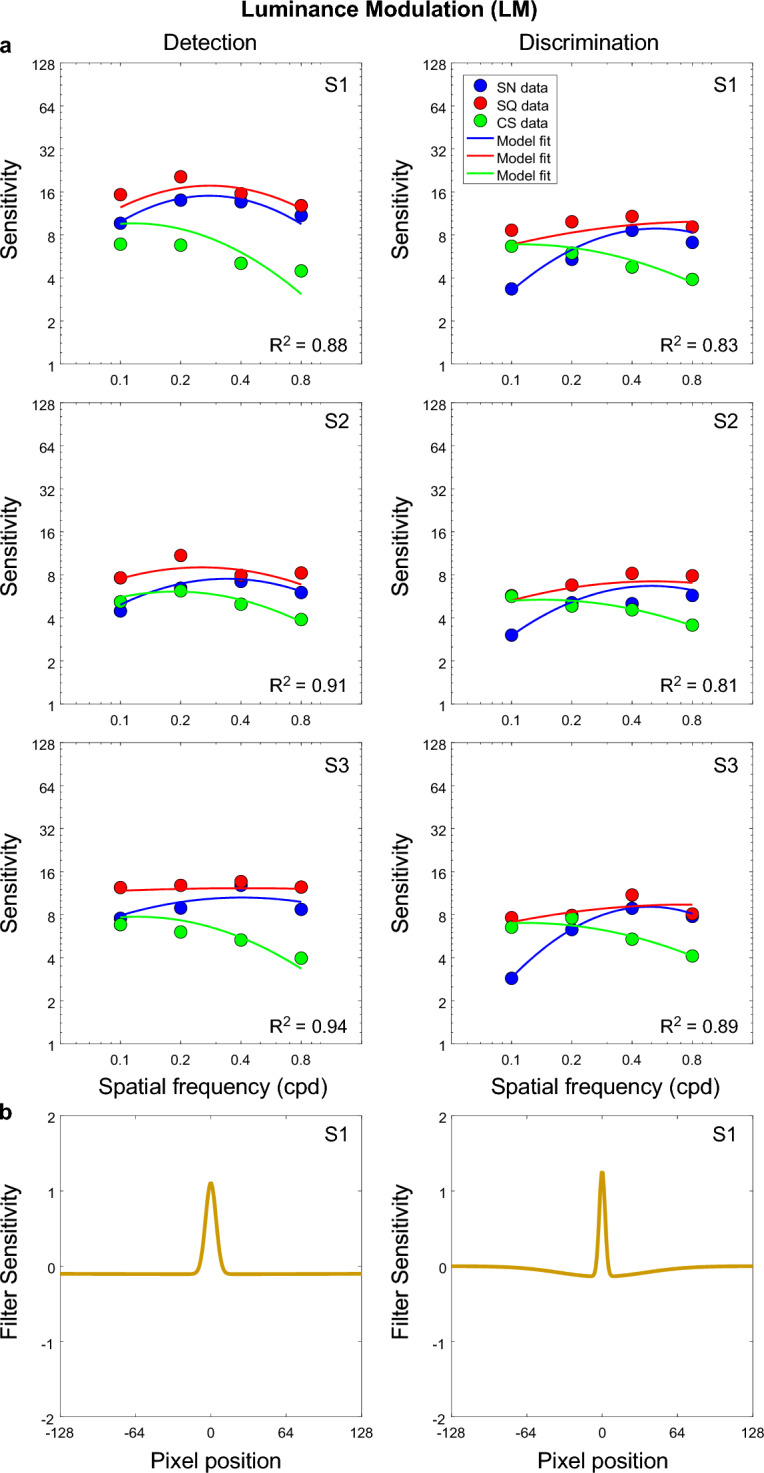
Luminance modulated texture: (**a**) Model fits (continuous lines) to sensitivity data (dots) for each triplet waveforms: sinewave (blue), square-wave (red) and cusp-wave (green) and (**b**) example corresponding shapes of the model filter (filter sensitivity) for one participant for both detection (left panels) and discrimination (right panels) tasks.

**Figure 9:**
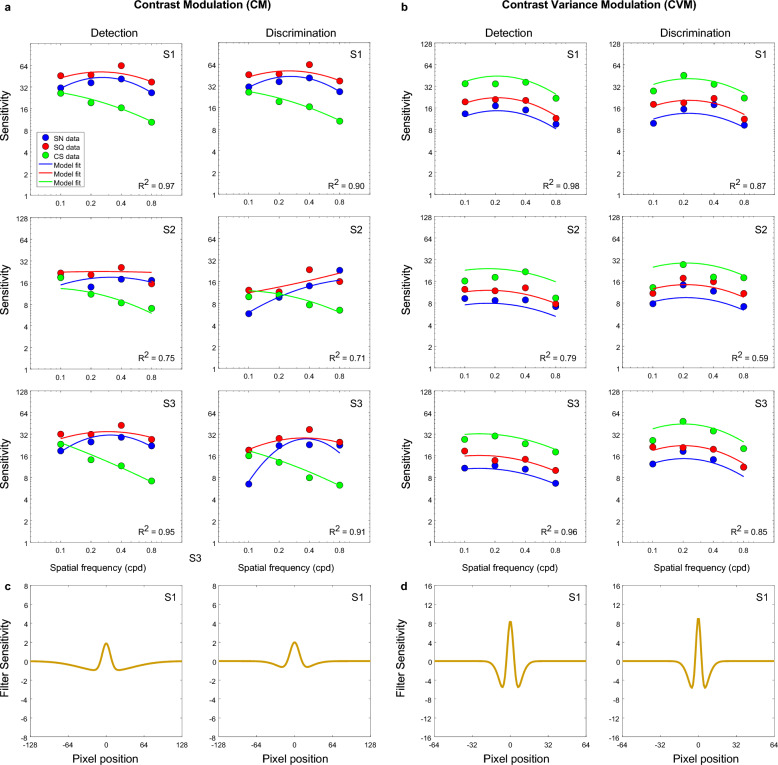
(**a**, **b**) Model fits (continuous lines) to sensitivity data (dots) for each triplet waveforms: sinewave (blue), square-wave (red) and cusp-wave (green) for the (**a**) contrast modulated, CM, and (**b**) contrast variance modulated, CVM, texture types for both detection (left panels) and discrimination (right panels) tasks. Example corresponding shapes of the model filter (filter sensitivity) for one participant for CM (**c**) and CVM (**d**) for detection (left panels) and discrimination (right panels) tasks.

The model fits are shown in Fig. 7 for OM and OVM textures, Fig. 8 for LM texture and Fig. 9 for CM and CVM texture, respectively. Overall, the model predicts reasonably well the ordering of sensitivities across spatial frequency and type of waveform. As a goodness of fit measure, we calculated the coefficient of determination R^2^ between model and data and these values are shown on the graphs. There is generally good agreement between model and data but inevitably the R^2^ values are lower when the data is mostly flat across SF, as for example in S1 and S2’s OM discrimination data, due to R being a measure of correlation, which is zero for flat data. The model fit for one condition, CVM, resulted in the lowest value of R^2^ ~ 0.4 and is not shown in Fig. 9b (see discussion below). From the CVM sensitivity data in Fig. 9b, one can see that the pattern of sensitivities for all three waveforms is very similar, varying only by a scaling factor.

For the CVM textures, data for the SN and SQ waveforms are similar in shape to the CS waveform but shifted towards lower sensitivity values, suggesting that data for all three types of waveform can be described by the same function subject to different scaling factors. Thus, for the CVM textures, we fitted the CS waveform with the DoG model and modelled the SQ and SN waveforms as 1/2 and 1/3 of the fitted DoG amplitude respectively. The resulting fits to CVM sensitivity data are shown in Fig. 9b. If energy depends on the intensity of the waveform, and intensity is proportional to the square of the amplitude, then the scaling factor for the SQ waveform suggests that if amplitude is reduced by half, then energy will be decrease fourfold.

The ‘perceptual’ receptive field profiles of the model filters are shown in Figs. 7, 8, 9 (lower panels) and the parameters of each are given in the Supplementary Information (see Table 1). As an indication of the size of the perceptual filter’s receptive field, the center and surround SDs are also given in degrees of visual angle (see Table 1 in the Supplementary Information), and were calculated as:

3 $$\text{Centre SD }\left(\text{deg}\right)=w\cdot\upgamma /\text{im}$$

4 $$\text{Surround SD }\left(\text{deg}\right)=w\cdot \delta \cdot \gamma /im$$

where *w*, the width of stimulus is 10 deg and $im,$ the image width used in Eq. 2 is 256. The receptive field of the ‘perceptual filter was estimated as 5 × Surround SD. The resulting values indicate that on average, across different texture types, the size of the perceptual RFs for the detection task (~ 17.4 deg) was about 3 times larger than that of the discrimination task (6.34 deg) (see Table 2 in the Supplementary Information).

**Figure 10:**
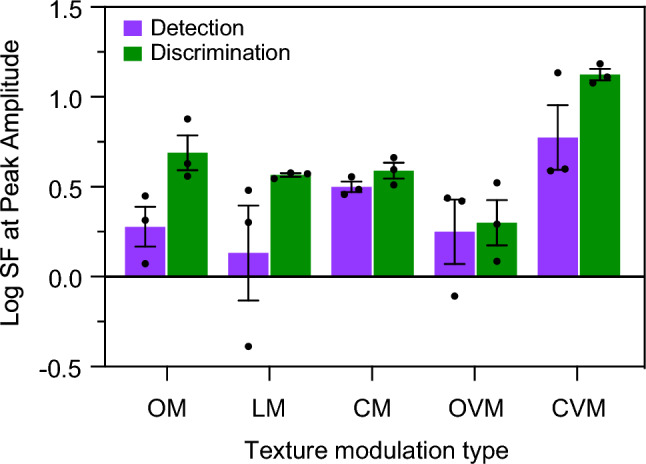
Filter model output. Individual (dots) and average across-participants (bars) analytical Log SF at peak amplitude of the filter model for both detection (magenta) and discrimination (green) tasks.

Finally, the individual and average across-participant analytical SFs at peak filter amplitude are shown in Fig. 10 for both detection (magenta) and discrimination (green) tasks. These modelling results show that for all types of textures, the analytical SF at peak amplitude is lower for detection than discrimination and is comparable across OM, LM, CM and OVM texture types, while for the CVM texture the SF at peak amplitude is higher.

Filter model output (Log SF at peak amplitude) differences were assessed with a two-way repeated-measures ANOVA with factors Task (detection vs. discrimination) and Texture type (OM, LM, CM, OVM, CVM). Greenhouse–Geisser correction was used where applicable. The analysis revealed a significant effect of task (F(1, 2) = 50.71, *p* = 0.019). The main effect of texture type (F(1.216, 2.432) = 5.899, *p* = 0.114) and the interaction effect between task and texture type (F(1.558, 3.116) = 0.945, *p* = 0.451) were not significant. Bonferroni-corrected post-hoc comparisons (paired-samples t-tests) revealed that all pairwise comparisons were not significant (all *p*’s > 0.066) except between LM and CVM for discrimination task (t(2) = 18.85, *p* = 0.028, 95% CI [− 0.976 − 0.141]).

## Discussion

We aimed to reveal whether the nature of the task determines whether region-based or edge-based mechanisms are involved in texture perception. Using five texture types (OM, OVM, LM, CM, CVM), three types of waveform (SN, SQ and CS) and four modulation spatial frequencies (0.1, 0.2, 0.4 and 0.8 cpd) our psychophysical data indicate that for the detection task, threshold amplitudes invariable followed the ordering SQ < SN < CS across all spatial frequencies. This ordering of thresholds follows that of the (inverse) of the relative root-mean-square (rms) energies of the waveforms with amplitudes of unity, which are respectively 1, 0.71 and 0.48, consistent with detection being mediated by a region-based mechanism that integrates texture energy across space. This finding is in keeping with previous OM texture studies reporting that the greater the mean orientation difference across the boundary the better the performance^22,23,29^. Psychophysical masking studies have suggested that the different types of texture modulation used in the present study are likely detected by different mechanisms^18,30^. Our finding that for each texture type, task and participant the same model successfully accounts for the results of all three varieties of waveform implies that these mechanisms are nevertheless agnostic to waveform type.

For the discrimination task at low texture spatial frequencies and with CVM textures at all spatial frequencies, the ordering of threshold amplitudes was CS ≤ SQ with both CS and SQ thresholds being smaller than SN. This suggests that the task employed a more edge-based mechanism. Our one-dimensional Difference of Gaussian (DoG) filter model fitted to the data showed that the SF of the filter at peak amplitude was lower for detection than discrimination across all texture types, with the exception of the CVM textures. Taken together with the psychophysical data, the model provides strong evidence that task requirements are important for whether edges or regions underpin texture processing.

### Models of texture perception

It is widely believed that texture processing can be modelled as a filter-rectify-filter, or FRF cascade (see reviews by Landy & Graham^19^, Graham^31^, and Victor et al.^32^). In the FRF model, the texture’s micropatterns are first detected by luminance-contrast-sensitive, or ‘1st-order’ band-pass filters. Filter outputs are then subject to a nonlinearity such as rectification or squaring in order to make all excursions from the mean level positive. The outputs of this stage are then pooled into ‘2nd-order’ band-pass filters whose responses encode the envelope modulation of the texture^3,29,33–39^.

Evidence that the 2nd-order envelope-tuned channels in the FRF scheme exist at multiple spatial scales comes from psychophysical studies using oblique masking of spatial-frequency-modulated textures^40^, subthreshold summation of orientation-modulated textures^24^ and single-unit recordings in cat cortical area 18 using contrast-modulated textures^41,42^. With this in mind, it is worth emphasizing that our single-DoG filter model, like that of the model described in Kingdom and Keeble^23^, should be considered as the computational equivalent of multiple spatial-frequency-tuned 2nd-order filters, analogous to the way that the luminance contrast sensitivity function represents the umbrella of sensitivities of multiple spatial-frequency-tuned luminance-contrast channels.

We find that with the same set of stimuli there is an apparent shift towards higher envelope spatial frequencies when the task changes from detection to discrimination. How might texture mechanisms be recruited for these different tasks? A similar set of stimuli was employed in both the detection and discrimination tasks, so this will likely lead to a similar outcome at the “bottom-up” task-independent stage One possibility therefore is that the task-dependent outcome is the result of a second stage of “top-down” processes that exploit prior knowledge of the processing requirements of each task and stimulus arrangement, with attention being directed to the relevant stimulus feature(s) (e.g., as has been argued for the perception of symmetry^43^ and motion^44^; for reviews see^45,46^). Specifically it has been suggested that the interaction between texture-sensitive mechanisms and the nature of the task reflects ‘biased-saliency’^32^ (see Fig. 2b in Victor et al.^32^). The idea here is that texture mechanisms are largely fixed, yet subject to the effects of prior knowledge that biases such properties as signal-to-noise ratios, filter sizes and filter gains, resulting in changes to the output decision. Our model is in keeping with the idea that average filter size is the property that is adjusted depending on the task.

### Supplementary Information

Supplementary Tables.

## Data Availability

All data are available online at: http://hdl.handle.net/11667/231.

## References

[1] Marr, D. Vision: A computational investigation into the human representation and processing of visual information. San Francisco: W. H.Freeman., ch.3, pp. 215–239 (1982).

[2] Julesz, B. Textons, the elements of texture perception, and their interactions. *Nature* **290**, 91–97 (1981). 7207603 10.1038/290091a0

[3] Malik, J. & Perona, P. Preattentive texture discrimination with early vision mechanisms. *J. Opt. Soc. Am. A* **7**, 923–932. 10.1364/josaa.7.000923 (1990). 2338600 10.1364/josaa.7.000923

[4] Li, A. & Zaidi, Q. Perception of three-dimensional shape from texture is based on patterns of oriented energy. *Vis. Res.* **40**, 217–242. 10.1016/s0042-6989(99)00169-8 (2000). 10793898 10.1016/s0042-6989(99)00169-8

[5] Li, A. & Zaidi, Q. Three-dimensional shape from non-homogeneous textures: carved and stretched surfaces. *J. Vis.* **4**, 860–878. 10.1167/4.10.3 (2004). 15595891 10.1167/4.10.3

[6] Cutting, J. E. & Millard, R. T. Three gradients and the perception of flat and curved surfaces. *J. Exp. Psychol. Gen.* **113**, 198–216 (1984). 6242750 10.1037/0096-3445.113.2.198

[7] Landy, M. S. Texture analysis and perception. . *In Werner, J. S. & Chalupa, L. M. (Eds.), The New Visual Neurosciences*, (Ch. 45, pp. 639–652). Cambridge, Mass.: MIT Press. (2013).

[8] Elder, J. H. & Sachs, A. J. Psychophysical receptive fields of edge detection mechanisms. *Vis. Res.* **44**, 795–813. 10.1016/j.visres.2003.11.021 (2004). 14967206 10.1016/j.visres.2003.11.021

[9] McIlhagga, W. Estimates of edge detection filters in human vision. *Vis. Res.* **153**, 30–36. 10.1016/j.visres.2018.09.007 (2018). 30291920 10.1016/j.visres.2018.09.007

[10] McIlhagga, W. H. & May, K. A. Optimal edge filters explain human blur detection. *J. Vis.* **12**, 9. 10.1167/12.10.9 (2012). 22984222 10.1167/12.10.9

[11] McIlhagga, W. & Mullen, K. T. Evidence for chromatic edge detectors in human vision using classification images. *J. Vis.* **18**, 8. 10.1167/18.9.8 (2018). 30208428 10.1167/18.9.8

[12] Nothdurft, H. C. Sensitivity for structure gradient in texture discrimination tasks. *Vis. Res.* **25**, 1957–1968. 10.1016/0042-6989(85)90020-3 (1985). 3832621 10.1016/0042-6989(85)90020-3

[13] Nothdurft, H. C. Texture segmentation and pop-out from orientation contrast. *Vis. Res.* **31**, 1073–1078. 10.1016/0042-6989(91)90211-m (1991). 1858322 10.1016/0042-6989(91)90211-m

[14] Gurnsey, R. & Laundry, D. S. Texture discrimination with and without abrupt texture gradients. *Can. J. Psychol.* **46**, 306–332. 10.1037/h0084319 (1992). 1451045 10.1037/h0084319

[15] Wolfson, S. S. & Landy, M. S. Examining edge- and region-based texture analysis mechanisms. *Vis. Res.* **38**, 439–446. 10.1016/s0042-6989(97)00153-3 (1998). 9536367 10.1016/s0042-6989(97)00153-3

[16] DiMattina, C. & Baker, C. L. Jr. Modeling second-order boundary perception: A machine learning approach. *PLoS Comput. Biol.* **15**, e1006829. 10.1371/journal.pcbi.1006829 (2019). 30883556 10.1371/journal.pcbi.1006829 PMC6438569

[17] Dakin, S. C. & Mareschal, I. Sensitivity to contrast modulation depends on carrier spatial frequency and orientation. *Vis. Res.* **40**, 311–329. 10.1016/s0042-6989(99)00179-0 (2000). 10793904 10.1016/s0042-6989(99)00179-0

[18] Schofield, A. J. & Georgeson, M. A. Sensitivity to modulations of luminance and contrast in visual white noise: separate mechanisms with similar behaviour. *Vis. Res.* **39**, 2697–2716. 10.1016/s0042-6989(98)00284-3 (1999). 10492831 10.1016/s0042-6989(98)00284-3

[19] Landy, M. S. & Graham, N. Visual perception of texture *in The Visual Neurosciences volume 169 Eds Chalupa, L. M. Werner, J. S. (Cambridge, MA: MIT Press), pp 1106–1118* (2004).

[20] Baker, C. L. Jr. Central neural mechanisms for detecting second-order motion. *Curr. Opin. Neurobiol.* **9**, 461–466. 10.1016/S0959-4388(99)80069-5 (1999). 10448168 10.1016/S0959-4388(99)80069-5

[21] Keeble, D. R., Kingdom, F. A., Moulden, B. & Morgan, M. J. Detection of orientationally multimodal textures. *Vis. Res.* **35**, 1991–2005. 10.1016/0042-6989(94)00284-s (1995). 7660604 10.1016/0042-6989(94)00284-s

[22] Kingdom, F. A., Keeble, D. & Moulden, B. Sensitivity to orientation modulation in micropattern-based textures. *Vis. Res.* **35**, 79–91. 10.1016/0042-6989(94)e0079-z (1995). 7839613 10.1016/0042-6989(94)e0079-z

[23] Kingdom, F. A. & Keeble, D. R. A linear systems approach to the detection of both abrupt and smooth spatial variations in orientation-defined textures. *Vis. Res.* **36**, 409–420. 10.1016/0042-6989(95)00123-9 (1996). 8746230 10.1016/0042-6989(95)00123-9

[24] Landy, M. S. & Oruc, I. Properties of second-order spatial frequency channels. *Vis. Res.* **42**, 2311–2329. 10.1016/s0042-6989(02)00193-1 (2002). 12220586 10.1016/s0042-6989(02)00193-1

[25] Prins, N. & Kingdom, F. A. Detection and discrimination of texture modulations defined by orientation, spatial frequency, and contrast. *J. Opt. Soc. Am. A* **20**, 401–410 (2003). 10.1364/JOSAA.20.000401 12630826

[26] Motoyoshi, I. & Kingdom, F. A. Differential roles of contrast polarity reveal two streams of second-order visual processing. *Vis. Res.* **47**, 2047–2054. 10.1016/j.visres.2007.03.015 (2007). 17555787 10.1016/j.visres.2007.03.015

[27] Schofield, A. J., Hesse, G., Rock, P. B. & Georgeson, M. A. Local luminance amplitude modulates the interpretation of shape-from-shading in textured surfaces. *Vision Res.* **46**, 3462–3482. 10.1016/j.visres.2006.03.014 (2006). 16650882 10.1016/j.visres.2006.03.014

[28] Prins, N. & Kingdom, F. A. A. Applying the model-comparison approach to test specific research hypotheses in psychophysical research using the palamedes toolbox. *Front. Psychol.* **9**, 1250. 10.3389/fpsyg.2018.01250 (2018). 30083122 10.3389/fpsyg.2018.01250 PMC6064978

[29] Landy, M. S. & Bergen, J. R. Texture segregation and orientation gradient. *Vision Res.* **31**, 679–691. 10.1016/0042-6989(91)90009-t (1991). 1843770 10.1016/0042-6989(91)90009-t

[30] Kingdom, F. A., Prins, N. & Hayes, A. Mechanism independence for texture-modulation detection is consistent with a filter-rectify-filter mechanism. *Vis. Neurosci.* **20**, 65–76. 10.1017/s0952523803201073 (2003). 12699084 10.1017/s0952523803201073

[31] Graham, N. V. Beyond multiple pattern analyzers modeled as linear filters (as classical V1 simple cells): Useful additions of the last 25 years. *Vision Res.* **51**, 1397–1430. 10.1016/j.visres.2011.02.007 (2011). 21329718 10.1016/j.visres.2011.02.007

[32] Victor, J. D., Conte, M. M. & Chubb, C. F. Textures as probes of visual processing. *Annu. Rev. Vis. Sci.* **3**, 275–296. 10.1146/annurev-vision-102016-061316 (2017). 28937948 10.1146/annurev-vision-102016-061316 PMC5629359

[33] Chubb, C. & Landy, M. S. Orthogonal distribution analysis: A new approach to the study of texture perception, in Computational Models of Visual Processing, (M. S. Landy and J. A. Movshon, eds.), Cambridge, MA: MIT Press, pp. 291–301. (1991).

[34] Chubb, C., Econopouly, J. & Landy, M. S. Histogram contrast analysis and the visual segregation of IID textures. *J. Opt. Soc. Am. A Opt. Image Sci. Vis.* **11**, 2350–2374. 10.1364/josaa.11.002350 (1994). 7931761 10.1364/josaa.11.002350

[35] Graham, N. Non-linearities in texture segregation. *Ciba Found Symp.* **184**, 309–322. 10.1002/9780470514610.ch16 (1994). 7882760 10.1002/9780470514610.ch16

[36] Sutter, A., Beck, J. & Graham, N. Contrast and spatial variables in texture segregation: Testing a simple spatial-frequency channels model. *Percept. Psychophys.* **46**, 312–332. 10.3758/bf03204985 (1989). 2798025 10.3758/bf03204985

[37] Sagi, D. Spatial filters in texture segmentation tasks. In B. Blum (Ed.), Channels in the visual nervous system: neurophysiology, psychophysics and models (pp. 397–424). London and Tel Aviv: Freund Publishing House. (1991).

[38] Motoyoshi, I. & Nishida, S. Cross-orientation summation in texture segregation. *Vision Res.* **44**, 2567–2576. 10.1016/j.visres.2004.05.024 (2004). 15358072 10.1016/j.visres.2004.05.024

[39] Kehrer, L. & Meinecke, C. A space-variant filter model of texture segregation: Parameter adjustment guided by psychophysical data. *Biol. Cybern.* **88**, 183–200. 10.1007/s00422-002-0369-3 (2003). 12647226 10.1007/s00422-002-0369-3

[40] Arsenault, A. S., Wilkinson, F. & Kingdom, F. A. Modulation frequency and orientation tuning of second-order texture mechanisms. *J. Opt. Soc. Am. A Opt. Image Sci. Vis.* **16**, 427–435. 10.1364/josaa.16.000427 (1999). 10069048 10.1364/josaa.16.000427

[41] Baker, C. L. Jr. & Mareschal, I. Processing of second-order stimuli in the visual cortex. *Prog. Brain Res.* **134**, 171–191. 10.1016/s0079-6123(01)34013-x (2001). 11702543 10.1016/s0079-6123(01)34013-x

[42] Mareschal, I. & Baker, C. L. Jr. Temporal and spatial response to second-order stimuli in cat area 18. *J. Neurophysiol.* **80**, 2811–2823. 10.1152/jn.1998.80.6.2811 (1998). 9862886 10.1152/jn.1998.80.6.2811

[43] Gheorghiu, E., Kingdom, F. A. A., Remkes, A., Li, H. O. & Rainville, S. The role of color and attention-to-color in mirror-symmetry perception. *Sci. Rep.* **6**, 29287. 10.1038/srep29287 (2016). 27404804 10.1038/srep29287 PMC4941524

[44] Lankheet, M. J. & Verstraten, F. A. Attentional modulation of adaptation to two-component transparent motion. *Vision Res.* **35**, 1401–1412. 10.1016/0042-6989(95)98720-t (1995). 7645269 10.1016/0042-6989(95)98720-t

[45] Carrasco, M. Visual attention: The past 25 years. *Vision Res.* **51**, 1484–1525. 10.1016/j.visres.2011.04.012 (2011). 21549742 10.1016/j.visres.2011.04.012 PMC3390154

[46] Scolari, M., Ester, E. F. & Serences, J. T. *Feature-and object-based attentional modulation in the human visual system. Oxford Handbook of Attention* (Oxford University Press, Oxford, UK, 2014).

