# Supplementary material for: Task-dependent contribution to edge-based versus region-based texture perception
Source: Sci Rep. 2024 Aug 2;14:17953. doi: 10.1038/s41598-024-68976-6 (PMC11297202; doi:10.1038/s41598-024-68976-6)
Supplement: Supplementary file 1 — Supplementary Tables. [file 41598_2024_68976_MOESM1_ESM.pdf]

Supplementary Information

Task-dependent contribution to edge-based versus region-based texture perception

Elena Gheorghiu[1,✉], Cassandra Diggiss[2] & Frederick A.A. Kingdom[2]

[1] University of Stirling, Department of Psychology, Stirling, FK9 4LA, Scotland, United Kingdom
[2] McGill Vision Research, Department of Ophthalmology, McGill University, Montreal, Canada
✉Corresponding author: elena.gheorghiu@stir.ac.uk

Table 1. Parameters of model filter fits to the data, for each type of texture, each task, and each participant. SD - standard deviation, DFT - discreet Fourier transform, FT - Fourier transform, SF - spatial frequency. Perceptual RF size = 5 x Surround SD in degrees.

Subject	Wave form	Task	Centre Gain (α)	Surround to Centre Gain Ratio (β)	Centre SD (γ)	Surround to Centre SD ratio (δ)	Centre SD (deg)	Surround SD (deg)	Perceptual RF size (deg)	DFT SF at Peak Amplitude	Analytical FT SF at Peak Amplitude
S1	OM	Detection	-0.33	1.526	6.07	7.1	0.2371	1.6835	8.4175	3	2.81
		Discrimination	0.09	0.828	2.50	11.85	0.0977	1.1572	5.786	4	4.26
	OVM	Detection	0.29	2.698	4.84	10.39	0.1891	1.9644	9.822	3	2.74
		Discrimination	-0.40	1.744	7.28	4.63	0.2844	1.3167	6.5835	3	3.33
	LM	Detection	13.49	9.252	4.43	107.63	0.1730	18.625	93.125	2	0.41
		Discrimination	8.68	1.467	2.46	14.98	0.0961	1.4395	7.1975	4	3.78
	CM	Detection	51.42	2.322	6.91	6.26	0.2699	1.6807	8.4035	3	2.87
		Discrimination	63.83	0.998	8.16	2.73	0.3187	0.8702	4.351	4	3.94
	CVM	Detection	148.51	1.296	2.90	2.23	0.1133	0.2526	1.263	14	13.61
		Discrimination	106.77	1.425	2.24	2.81	0.0875	0.2459	1.2295	15	15.26
S2	OM	Detection	-0.10	0.738	3.06	42.69	0.1195	5.1028	25.514	2	1.18
		Discrimination	-0.07	0.849	0.96	19	0.0375	0.7125	3.5625	8	7.55
	OVM	Detection	0.23	2.656	5.66	8.99	0.2211	1.9876	9.938	3	2.64
		Discrimination	-0.23	1.530	5.18	13.51	0.2023	2.7337	13.6685	2	1.96
	LM	Detection	6.34	2.793	1.53	56.55	0.0598	3.3797	16.8985	2	2.01
		Discrimination	1.705	1.670	26.16	0.0245	1.0219	0.0250	0.125	4	3.51

	CM	Detection	21.94	2.558	5.17	8.35	0.202	1.6863	8.4315	3	3.01
		Discrimination	18.10	1.677	3.51	7.73	0.1317	1.0599	5.2995	5	4.6
	CVM	Detection	32.48	3.471	2.67	13.89	0.1043	1.4487	7.2435	1	3.97
		Discrimination	58.81	1.608	2.09	3.95	0.0816	0.3225	1.6125	13	12.95
S3	OM	Detection	0.38	0.569	6.62	8.08	0.2586	2.0894	10.447	2	2.06
		Discrimination	-0.15	1.050	3.71	9.15	0.1449	1.326	6.63	4	3.62
	OVM	Detection	0.36	2.038	6.59	30.86	0.2574	7.944	39.72	2	0.78
		Discrimination	0.34	1.659	5.53	22.18	0.2160	4.7912	23.956	2	1.22
	LM	Detection	-10.57	2.635	3.3	14.59	0.1289	1.8807	9.4035	3	3.02
		Discrimination	8.42	1.286	2.01	19.05	0.0785	1.4957	7.4785	4	3.74
	CM	Detection	39.75	0.634	6.05	4.27	0.2363	1.0091	5.0455	4	3.59
		Discrimination	29.58	1.070	5.57	6.24	0.2176	1.3577	6.7885	3	3.24
	CVM	Detection	42.22	2.471	2.52	14.86	0.0984	1.4628	7.314	4	3.88
		Discrimination	126.24	1.432	2.98	2.66	0.1164	0.3096	1.548	12	11.94

Table 2. Average across-participants size of the predicted perceptual filter's receptive field for the detection and discrimination task. When averaged across types of textures, this was about 3 times larger for the detection (~ 17.4 deg) compared to discrimination (6.34 deg) task.

	Detection	Discrimination	Average
OM	14.79	5.326	10.06
OVM	19.83	14.74	17.28
LM	39.81	4.934	22.37
CM	7.294	5.480	6.387
CVM	5.274	1.463	3.368
Average	17.40	6.388	11.89
